# New Insights into Cockroach Control: Using Functional Diversity of *Blattella germanica* Symbionts

**DOI:** 10.3390/insects11100696

**Published:** 2020-10-13

**Authors:** Xiaoyuan Pan, Xuejun Wang, Fan Zhang

**Affiliations:** 1 Key Laboratory of Animal Resistance Biology of Shandong Province, College of Life Science, Shandong Normal University, 88 East Wenhua Road, Jinan 250014, China; panxiaoyuan2018@163.com; 2 Shandong Center for Control and Prevention, 16992 Jingshi Road, Jinan 250014, China

**Keywords:** *Blattella germanica*, symbionts, interaction, pest control

## Abstract

**Simple Summary:**

Insect hosts have close relationships with microbial symbionts. The limited metabolic networks of most insects are enhanced by these symbiotic relationships. Using symbiotic microorganisms for biological control of insects and insect-borne diseases has become an important research topic and shows potential for the development of applicable control approaches. *Blattella germanica* (L.) is public health pest worldwide; it is difficult to control because of its strong reproductive ability, adaptability, and resistance to insecticides. In this paper, the diverse biological functions (nutrition metabolism, reproductive regulation, insecticide resistance, defense, and behavior management) of symbionts, their interaction mechanism with hosts, and the research progress in the control of *B. germanica* are reviewed and discussed.

**Abstract:**

Insects have close symbiotic relationships with several microbes, which extends the limited metabolic networks of most insects. Using symbiotic microorganisms for the biological control of pests and insect-borne diseases has become a promising direction. *Blattella germanica* (L.) (Blattaria: Blattidae) is a public health pest worldwide, which is difficult to control because of its strong reproductive ability, adaptability, and resistance to insecticides. In this paper, the diverse biological functions (nutrition, reproductive regulation, insecticide resistance, defense, and behavior) of symbionts were reviewed, and new biological control strategies on the basis of insect–symbiont interaction were proposed. We highlight new directions in *B. germanica* control, such as suppressing cockroach population using *Wolbachia* or paratransgenes, and combining fungal insecticides with synergistic agents to enhance insecticidal efficacy.

## 1. Introduction

Cockroaches are an ancient group representing one of the most successful life forms. They have high adaptability to a wide range of habitats and environmental conditions from Arctic cold to tropical heat [1]. There are approximately 4500 species of cockroaches worldwide, among which about 30 species often coexist with human populations and few are considered indoor health pests [2,3]. Recently, cockroach infestations have been increasing across the world. The German cockroach, *Blattella germanica*, which ordinarily resides in human habitats, is a common domestic pest species of economic and medical importance [4]. A recent study showed that the global mean infestation trend of *B. germanica* in human dwelling ranged from 40% to 70% [5]. *B. germanica* has an elaborate social structure that includes kin recognition, information transfer, common shelter, and social dependence [6]. They are omnivorous, and only very little food is needed to sustain large populations. The feeding mechanisms and filthy breeding habits of cockroaches make them ideal carriers and transmitters of a variety of pathogens such as pathogenic bacteria, fungi, viruses, and helminths mechanically and occasionally biologically [7,8,9,10]. Additionally, their feces, debris, and secretions can cause serious allergic reactions in humans [11]. Insects including cockroaches harbor a large number of symbiotic microorganisms; these symbionts have coevolved with the host insect, thereby forming a complex and dynamically balanced microecosystem. They play an irreplaceable role in insect evolution and adaptation and are the health guardians of host insects. Studies have shown that the microbial biomass in insects exceeds the number of cells of the insect itself, and the biomass can reach 1–10% of the dry weight of insects. From this perspective, insects are actually a multi-species complex [12]. Insects provide a relatively stable living environment and nutrient resources for symbionts, while symbionts are also involved in many life activities of insects, including providing essential nutrients, digesting indigestible food components, regulating the immune system to resist pathogens and parasites, secreting bioactive substances against predators and parasitoids, and participating in intraspecific and interspecific information transfer [13,14]. Therefore, symbiotic microbes are referred to as a multifunctional organ of insect hosts, which play an important role in regulating various physiological functions of insects and maintaining intestinal homeostasis. For some insect vectors that spread diseases, symbiotic microbes can affect the vector efficacy and development time of host insects [15,16,17], showing the application potential of symbiotic microbes in insect-borne infectious disease control. For example, the vital immune component AsSRPN6 can be induced to be expressed by *Enterobacter cloacae*, which can inhibit *Plasmodium falciparum* development in *Anopheles stephensi* [18].

Since the discovery of *Blattabacterium* sp. [19], the symbiont harbored in cockroaches, symbiotic microbes have been found in a wide range of insects. On the basis of their morphology or their life histories, the symbiotic microbes in insects can be roughly divided into five categories: bacteria, fungi, archaea, viruses, and protozoa, of which bacteria are found in almost all insect guts and are often most abundant [14,20]. Additionally, they can also be divided into two categories on the basis of differences in symbiotic modes: endosymbiont and ectosymbiont. Endosymbionts are the microbes that live inside insect tissue cells, whereas ectosymbionts live outside insect cells. Endosymbionts, such as *Blattabacterium* and *Wolbachia*, usually live in the Malpighian tubules, fat bodies, blood cells, and ovarioles of insects [21,22]. Ectosymbionts include the microorganisms that dissociate in the gut cavity or attach to the insect gut wall cells, which are aggregated in the digestive tract in the form of gut microbiota. The gut flora plays a particularly important role in the catabolic process of insects, and the insect gut also provides a stable living environment for these intestinal microbes. Depending on the degree of interdependence between microbes and their hosts, symbiotic microbes can be divided into primary symbionts (i.e., obligate symbionts) and secondary symbionts (i.e., facultative symbionts). Primary symbionts can be vertically transmitted from generation to generation and have long-term coevolutionary relationships with the host insect, most of which are closely related to the survival, reproduction, and evolution of hosts [23]. Compared with the primary symbionts, the coevolutionary relationships between secondary symbionts and the host are relatively short, and they are mainly related to the adaptability of the host. Secondary symbionts are able to colonize new hosts with low-level horizontal transmission, although vertical transmission occurs occasionally [24].

**Figure 1:**
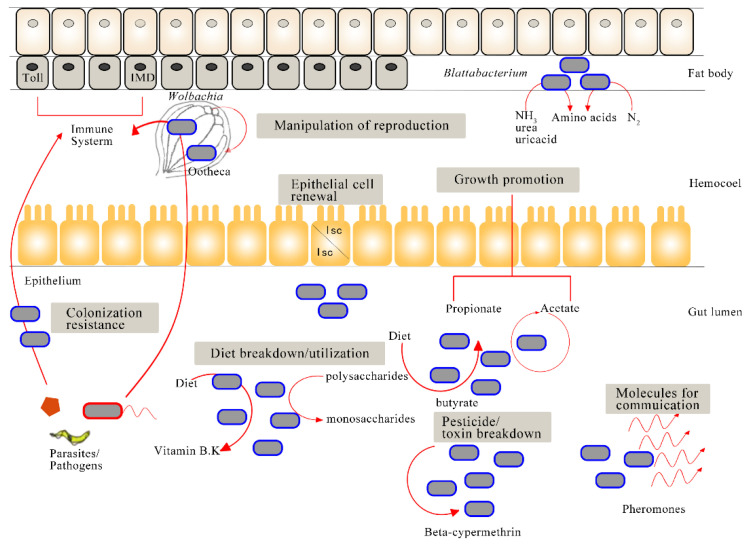
Hypothesized functions of the symbionts of *Blattella germanica*. The abundant gut symbiotic microbes have been demonstrated to be involved in nutritional physiology, reproductive regulation, pesticide resistance, and immune defense [23]. A prime example for nutritional physiology is that symbionts can be involved in the nitrogen cycle of the host and provide riboflavin and other vitamins, such as *Lachnospira* in the host’s gut, synthesizing acetate, propionate, and butyrate [32,39]. Moreover, symbionts can activate the immune system of the host to defend against pathogens, such as *Pseudomonas* and *Bacillus*, and can strongly inhibit the growth of *Beauveria bassiana* in vitro [33,40,41]; the endosymbiont *Wolbachia* might regulate the reproduction of *B. germanica* [30]. In addition, gut bacteria can synthesize pheromones (e.g., volatile carboxylic acids) or interstitial hormones [42].

Over the past few decades, the widespread and overuse of chemical insecticides has led to a growing phenomenon of pesticide resistance in cockroaches, especially in *B. germanica*; meanwhile, it has resulted in environmental pollution and adverse effects on other organisms in the ecosystem [25,26,27,28]. This is especially the case with the German cockroach [29]. Resistance in the *B. germanica* population has become a substantial problem that causes control failures in many areas of China. Therefore, there is an urgent need to develop novel cockroach control strategies. There are abundant symbiotic microbes that can assist the host’s complete life activities including growth, development, and reproduction in the cockroaches (Table 1; Figure 1). In view of the important biological functions of symbiotic microorganisms in insects, the study of their potential application in pest control has attracted much attention in recent years. In this review paper, we elucidate the diverse biological functions (nutrition metabolism, reproductive regulation, insecticide resistance, defense, and behavior management) of symbionts, their interaction mechanism with hosts, and the research progress in the control of *B. germanica*. We highlight new directions in controlling *B. germanica*, such as utilizing *Wolbachia* to manipulate host reproduction to suppress the pest population or promoting applications of entomopathogenic fungi by disturbing the microecological balance of cockroach gut microbiota with a gel bait synergy agent. Especially for vector insects, paratransgenes could reduce the insect’s vector capacity by interfering with the development of the pathogen within the insect.

**Table 1:** The category, distribution, and function of some important symbiotic bacteria of cockroach.

Bacteria	Category	Distribution	Function	Reference
*Blattabacterium*	Flavobacteriales	A special cell of fat body	Participate in nitrogen assimilation, uric acid degradation, and nutrient provisioning	[19]
*Wolbachia*	Proteobacteria	Reproductive tissues, digestive tract, thorax, abdomen, salivary gland, etc.	Reproductive regulations (e.g., cytoplasmic incompatibility)	[30]
*Salmonella* spp.	Proteobacteria	Gut	Increase the host drug resistant	[10]
*Bacteroides*	Bacteroidetes	Gut	Carbohydrate metabolism and transport; assist the host defense; entomopathogenic fungi	[31]
*Lachnospira*	Firmicutes	Gut	Hydrolyze polysaccharide; assist digestion; synthesize acetate, propionate, and butyrate	[32]
*Pseudomonas*	Proteobacteria	Gut	Secrete versatile secondary metabolites; provide protection from parasites and pathogens	[33]
*Bacillus*	Bacteriophyta	Gut	Inhibit microbial growth by secreting antifungal compounds and antibiotic-like compounds	[34]
*Weissella*	Firmicutes	Gut	Produce many antimicrobial agents such as adhesion inhibitors, organic acids, and bacteriocins	[35]
*Rickettsia*	Proteobacteria	Digestive organs, salivary glands, reproductive organs, etc.	Participate in reproductive regulation; increase host resistance	[36]
Acetobacteraceae	Proteobacteria	Gut	Participate in carbohydrate fermentation and lactate metabolism	[37]
*Providencia*	Proteobacteria	Gut	Assist the host defense natural predators	[10]
*Fusobacterium*	Fusobacteria	Gut	Ferment both glucose and amino acids	[32]
*Enterococcus* sp.	Firmicutes	Gut	Anti-phytopathogenic fungal activity	[38]

## 2. Nutrition and Development: Reducing Survivability

Insects provide stable habitats and nutrition for symbionts, and, in return, symbionts can assist the host in feeding and digestion, thereby expanding the range of the host’s diet and even altering the eating habits of the host [39]. Symbionts can produce various digestive enzymes and participate in the nitrogen cycle of the host, which can accelerate metabolism and transformation of nutrients. As an omnivorous insect, the German cockroach harbors a variety of symbiotic microorganisms, which can provide a considerable amount of riboflavin and other vitamins that are conducive to a nutritionally balanced diet. Additionally, *Lachnospira* in the host’s gut can synthesize acetate, propionate, and butyrate; Bacteroides are capable of hydrolyzing polysaccharides and have the ability to transform complex polysaccharides into monosaccharides that can be utilized for further digestion and absorption by the host [32]. Cockroaches can also store surplus nitrogen in urine cells of the fat body in the form of uric acid crystals so that when the quantity of nitrogen in food is limited, it can be supplemented by the uric acid. There is a type of bacteriocyte living around the urine cells, within which there is a large number of *Blattabacterium*. According to genomic sequencing analysis, *Blattabacterium* can synthesize most amino acids using a small amount of substrate (e.g., glutamate, urea, ammonia); thus, they are capable of making the most of the limited food supply and metabolites to provide *B. germanica* with almost all of the amino acids it needs, as well as certain vitamins [19]. Indeed, this is one of the main reasons for the widespread distribution and strong vitality of the German cockroach.

Studies showed that, after the removal of gut microbes, the egg pod produced by female adults of *B. germanica* appeared sunken and wizened, the hatching rate of larvae decreased, and the proportion of female offspring in the population also decreased [43,44]. Compared with that of the normal *B. germanica*, the fecundity of *B. germanica* without gut microbes showed a downward trend, showing an adverse effect on reproduction [43,44]. In addition, after treatment with antibiotics, the mortality of *B. germanica* larvae was higher and the growth period from larva to adult was longer [43,44], which suggests that the absence of some important endosymbionts might reduce or even hinder the efficiency of the nutritional metabolism to affect growth and development of host insect. Moreover, beta-cypermethrin-resistant cockroaches (R strain) had longer nymphal developmental periods and shorter adult longevities than susceptible cockroaches (S strain). The analysis of gut microbiota composition revealed that the relative abundances of *Lactobacillus* and an unclassified Acetobacteriaceae in the foregut and midgut of R strain were significantly lower than in S strain [45]. *Lactobacillus* and Acetobacteraceae are involved in nutrient absorption and metabolism of host insects, such as degradation of carbohydrates and lactic acid metabolism [14,37,46], which explains the growth and development retardation of the R strain. However, the specific mechanism still remains to be elucidated. This suggests that using antibiotics to remove gut symbiotic bacteria could decrease cockroaches’ survivability, especially in reproduction and development, and even influence activity of metabolic enzymes to reduce allergen secretion.

## 3. Reproductive Regulation: Potential Population Suppress Strategy Using *Wolbachia*

In the insects’ reproductive tissues, there are also many symbiotic microbes such as *Wolbachia*, *Cardinium*, and *Spiroplasmas*. Generally, these symbionts are vertically transmitted through the eggs; they can regulate host’s reproduction via some special mechanisms, thereby increasing the efficiency of their maternal transmission among hosts [47].

*Wolbachia*, which belongs to α-proteobacteria, is a maternally inherited bacterial symbiont widespread in many species of insects including cockroaches (*Blattella* sp. and the *Supella longipalpa*) [30,48]. A highly homologous *Wolbachia* surface protein (wsp) gene fragment was successfully amplified from the genomic DNA of the adult German cockroaches, implying that *Wolbachia* may be widespread in *B. germanica* [49]. *Wolbachia* can manipulate host reproduction in a variety of ways, including parthenogenesis, male killing, feminization, and, most commonly, cytoplasmic incompatibility (CI) [50]. UniCI (unidirectional CI) is commonly expressed as embryonic lethality when *Wolbachia*-infected males mate with uninfected females, with all other mating being fertile. Conversely, crosses between individuals containing different types of *Wolbachia* will cause infertility (bidirectional CI, biCI) [51]. Studies on *Wolbachia*-insect associations showed that CI could allow *Wolbachia* strains, such as *w*MelPop strain, to invade mosquito populations even though they confer a fitness cost such as increased mortality [52]. In addition, compared to *Wolbachia*-infected *Trichogramma karkai*, uninfected *T. karkai* have a higher reproductive rate and their eggs develop faster [53,54]. It is worth noting that the presence of *Wolbachia* can protect insects from pathogens and limit their ability to transmit many insect-borne pathogens, including dengue virus, yellow fever virus, Chikungunya virus, and plasmodium [55,56]. *Cardinium* and *Spiroplasmas* are recently discovered symbiotic bacteria that can lead to reproductive abnormalities in the host, which are functionally similar to those produced by *Wolbachia* [57,58]. The distribution ranges of *Cardinium* and *Spiroplasmas* are limited in arthropods, while neither infection type has been found in cockroaches.

Population suppression and population replacement programs using *Wolbachia*-induced CI are particularly attractive due to the ability of *Wolbachia* to manipulate insect reproduction and to interfere with major human pathogens. Population suppression is a method analogous to the use of microbial pesticides to control vector insects. For mosquitoes, by continuously releasing *Wolbachia*-carrying males to mate with nature females to induce CI, their offspring will die during the embryonic period, thereby controlling the number of populations below a critical infection threshold that can trigger an epidemic of disease [59,60,61]. Population replacement is a method similar to vaccinating vector insects. *Wolbachia*-infected females can produce *Wolbachia*-carrying offspring after mating with either infected or uninfected males [62,63]. Female mosquitoes, which carry the *Wolbachia* strain that are resistant to human pathogens, were released into the natural population, eventually realizing disease-resistant mosquito replacement after enough passage, thereby reducing or even blocking the transmission of vector-borne pathogens [63]. Either strategy requires the establishment of insect strains infected with *Wolbachia* or introduction of a new *Wolbachia* strain [64,65]. Currently, embryonic microinjection can be used to transfer *Wolbachia* within the same or different populations and generate new *Wolbachia* infection types by transferring infected cytoplasm between eggs [65]. For example, a stable infection of *w*Mel in tetracycline-cured *A. albopictus* was generated using embryonic microinjection, and then biCI occurred in crosses between these single-infected mosquito strains and wild dual-infected mosquitoes; thus, this mosquito population could completely interrupt dengue transmission [66]. Furthermore, adult female and male *Aedes aegypti* were released at Gordonvale and Yorkeys Knob sites in early January 2011, with a total of 157,300 (Gordonvale) and 141,600 (Yorkeys Knob) adults released. According to the monitoring results, *A. aegypt*i had maintained more than 90% *Wolbachia* infection frequencies in both locations at 5 weeks after release. One year later, virus-blocking and *Wolbachia* density phenotypes persisted in *w*Mel-infected *A. aegypti* [67].

Given the mechanisms of *Wolbachia*–host interactions, population suppression has the potential to become a new potential prevention and control technology for cockroaches. *Wolbachia* could also be used to control the reproduction of German cockroach in a similar manner to releasing males. Major procedures include mass rearing, sex separation, investigation of CI in natural populations, *Wolbachia* microinjection, mating confirmation, competition, packaging and transportation, release, and field monitoring [68]. For example, we could find a strain of *Wolbachia*, which could induce CI and has high reproductive capacity, from the cockroach species that naturally carry *Wolbachia* (e.g., *S. longipalpa*). Then, we could inject this *Wolbachia* into the cells of German cockroaches for serial passage in vitro. After that, we could inject the *Wolbachia* that has the ability to infect *B. germanica* into the ootheca or embryos of cockroaches for the maternal transmission. The male offspring infected with *Wolbachia* would be artificially sent into fields for copulating with uninfected female cockroaches. These female cockroaches would produce the progenies that develop abnormally or die during the embryonic stage on account of the effect of *Wolbachia*-mediated CI. 

The cockroach control strategies using *Wolbachia* might face public and technological challenges, such as increased potential risk of allergen and pathogen transmission, long life spans of cockroaches compared to other insects (i.e., mosquitoes), and establishment of new cockroach populations with *Wolbachia*. It is worth mentioning that some species of cockroach (e.g., *Periplaneta americana*, *Blatta orientalis*, and *Nauphoeta cinerea*) exhibit thelytoky, a type of parthenogenesis in which female offspring are produced without fertilization [69]. The existence of the parthenogenesis phenomenon may affect the practical application effect of the *Wolbachia*-based suppression approach, because cockroaches could produce offspring without mating. However, this phenomenon has not been discovered in *B. germanica* [70]. In addition, mass releases of male cockroaches, which have a long life span, can also act as nuisance pests and disease vectors. McMeniman et al. [15] successfully introduced a life-shortening *Wolbachia* strain, *w*MelPop, from *Drosophila melanogaster* into the mosquito vector, *A. aegypti*, to halve adult life span under laboratory conditions, thereby reducing pathogen transmission. If such a *Wolbachia* strain with the ability to induce CI and life-shortening could be transferred into *B. germanica* after continuous serial passage, the effect and feasibility of cockroach population suppression would be greatly enhanced. This *Wolbachia* strain might make their offspring die in the embryonic period and simultaneously shorten the lifespan of the cockroach, thereby reducing the cockroach population and the risk of pathogen transmission. In the long run, this may be an effective method. However, in the future, its feasibility still needs to be proven through rigorous experiments in laboratory and a large number of field simulation tests, so as to predict the speed and practical effect of population substitution. Furthermore, more in-depth studies and the assessment of epidemiological and entomological risks before the registration of related biocontrol products must be performed to persuade the public to accept this new approach.

## 4. Resistance to Pesticides: Symbiont-Mediated Potential Direction of Resistance Management

Currently, the abuse of insecticide causes pests to develop insecticide resistance, which has become the main barrier to pest control [71,72]. German cockroaches are resistant to major classes of insecticides currently in use (e.g., organochlorine, organophosphorus, carbamate, and pyrethroid insecticides) perhaps due to the shorter life cycle and stronger fertility of the German cockroach as compared to other cockroach species [25,73,74,75]. Previous studies on insecticide resistance mechanisms largely focused on the following aspects: target site insensitivity, increased metabolic detoxification, and decreased penetrability of the epidermis [72,76]. However, in recent years, the vital contribution of symbionts to the insecticide resistance of insects has gradually become the research hotspot. Many types of symbionts can help insects degrade pesticides and confer host resistance [77]. The long evolutionary history of endosymbionts enables them to keep their hosts alive to their mutual advantage. For instance, the fenitrothion-degrading *Burkholderia* confers its host the bean bug *Riptortus pedestris* (Hemiptera: Coreidae) resistance to fenitrothion [78]. Similarly, in *Bactrocera dorsalis* (Diptera: Tephritidae), the phosphatase hydrolase genes of the gut symbiont *Citrobacter* sp. were highly expressed when trichlorphon existed, which could help host degrade trichlorphon [79]. Thus, it is necessary to consider the role of host symbionts in pesticide resistance during the implementation of cockroach control measures.

There are two main mechanisms of symbiont-mediated resistance to insecticides. Firstly, symbionts can be induced by pesticides to produce detoxification enzymes that are able to degrade insecticides via cometabolism or mineralization. For instance, one kind of symbiotic yeast in *Lasioderma serricorne* (F.) (Coleoptera: Anobiidae) can directly produce two hydrolytic enzymes to degrade flavone, resorcinol, or tannic acid in order to assist the host in metabolic detoxification [80]. Compared with susceptible *Candida lipolytica* strains isolated from *Nilaparvata lugens* (Homoptera: Delphacidae), the activities of detoxifying enzymes such as carboxylesterase (CarE) and mixed-function oxidase (MFO) in the imidachloprid-resistant strain were significantly increased, indicating that the detoxifying enzyme activities of the symbionts could be activated or enhanced by pesticides to help hosts decompose toxic substances [81]. Secondly, the interaction between gut microbiota (symbionts) and the insect immune system leads to the enhancement of insecticide resistance. For example, the gut bacterium *Enterococcus* sp., vitamin C, and acetylsalicylic acid may protect the immune system of *Plutella xylostella* (Lepidoptera: Plutellidae) from the damage caused by the pesticide chlorpyrifos, thereby contributing to the pesticide resistance. In contrast, *Serratia* sp. reduced the resistance to chlorpyrifos [82]. Additionally, symbionts have the ability to degrade other exogenous materials, such as toxic secondary metabolites produced by plants, including alkaloids and phenols, to help the host resist the defense system of plants. For example, the dominant gut microbes *Pseudomonas* and *Serratia* of *Dendroctonus ponderosae* (Coleoptera: Scolytidae) can utilize metabolic pathways for degrading terpenes [83]. The complete metabolic pathway of aerobic degradation of catechol is also found in the gut microbiota of *P. xylostella* [84].

A recent study confirmed that gut microbiota plays an important role in *B. germanica* insecticide resistance. After treatment with antibiotic, the indoxacarb-resistant strain of the German cockroach, of which the gut microflora differs greatly from that of the susceptible strain, increased susceptibility to indoxacarb. The resistance of the susceptible strain cockroach increased after the gut microbes of the resistant strain were transferred to the susceptible strain through fecal transplant. The disruption of the gut microbiota affected reproductive life-history traits which could enhance resistance at the German cockroach population level [85]. According to experiments carried out in our laboratory using the Miseq second-generation sequencing technology, there are significantly different compositions of gut microbiota between the beta-cypermethrin-resistant (R) strain and the susceptible (S) strain of *B. germanica*. Their dominant microorganisms were roughly identical, but there was great difference in the microbial abundance. In compared to the S strain cockroaches, the relative abundances of *Lactobacillus* and an unclassified Acetobacteriaceae in the gut of R strain were significantly lower, but the relative abundances of *Parabacteroides* and *Weissella* were obviously higher [45]. *Parabacteroides* and *Weissella* can be involved in the degradation of complex compounds [86,87]. Enzyme activity tests further revealed that CarE and glutathione *S*-transferase (GST) activities were enhanced after the *Enterobacter* sp. and *Weissella* were transplanted back into the gut of *B. germanica*, suggesting that pesticide resistance may be related to the difference in composition and abundance of gut microbiota between R and S strains (manuscript in preparation). Our results advanced understanding of an underlying mechanism of *B. germanica* insecticide resistance and found the vital bacterial strains that may contribute to degrade pesticides from the German cockroach gut. We highlighted a new potential direction for resistance management of *B. germanica*; by interfering with critical strains from gut microbiota which are related to enhanced resistance, the pesticide resistance of cockroaches is weakened, thus increasing the insecticidal efficacy. In the future, we can culture these strains in vitro to degrade chemical pesticides and control environmental pollution. Furthermore, the gut flora of resistant cockroaches might be replaced by that of conventional cockroaches via fecal transplants, thus reducing pesticide resistance of cockroach population.

## 5. Immune and Defense: Improving the Effect of Biological Control Using Pathogenic Microorganisms

Insects face a variety of survival pressure in the natural environment, including the attack of natural enemies, pathogen and parasite infection, and high temperature and cold stress. Symbionts play an indispensable role in insects’ fight against these threats. Many studies showed that aseptically treated insects are more susceptible to pathogen and parasite infection than the control group [88,89]. For social insects such as cockroaches, the probability and the risk of pathogens spreading among individuals are significantly increased. Symbionts provide protection for host insects, such that the symbiotic combination has greater competitive advantages, which is conducive to the stable maintenance of symbiotic relationship.

Symbionts are able to activate the host’s innate immune system to induce the production of antimicrobial peptides to impact host pathogen proliferation. By comparing the expression profiles of immune genes of the axenic and nonaxenic mosquitoes, it was found that the presence of gut microbiota could activate the immune system of *Anopheles stephensi* and upregulate the expression levels of immune genes such as cecropin, defensin, and gambicin [90]. The symbionts in *Apis mellifera* can induce the host bee to produce antibacterial peptides and defensin to enhance immunity [91]. *Spiroplasma* can also improve the sensitivity of *D. melanogaster* to certain pathogens [92]. Additionally, symbionts can produce toxic metabolites to directly protect hosts. The symbiont *Pseudomonas* of the rove beetles *Paederus sabaeus* can produce pederin, a kind of potent polyketide toxin, to prevent hosts from being preyed on by wolf spiders [93,94]. In the aphid *Acyrthosiphon pisum*, its symbiotic bacteria *Hamiltonella defensa* can protect it from the parasitoid wasp *Aphidius ervi* through producing toxins to directly kill the wasp larvae [95]. The gut symbiotic bacteria *Bacillus subtilis* of honeybees can synthesize surfactin for defending against the pathogen *Paenibacillus larvae* [96,97]. Furthermore, symbionts can also indirectly protect insect hosts by protecting important third parties. Fungus-growing ants have a unique ability to select and cultivate certain fungi with a lack of competitiveness as food sources. The actinobacteria (*Pseudonocardia* spp.) from the ant *Apterostigma dentigerum* can produce the antifungal substance dentigerumycin to selectively kill a parasitic fungus (*Escovopsis* sp.), thereby protecting the symbiotic relationship between the ants and the fungus they cultivate for food [98]. Body color is an importantly ecological identifying characteristic associated with predatory insects. Insect symbionts can control the body color of insects to protect them from predators. In the pea aphid *Acyrthosiphon pisum*, the symbiont *Rickettsiella* can induce aphids to synthesize green pigments and change their body color from red to green, which helps them avoid being preyed upon by predators that tend to prey on red aphids, such as ladybird beetles [99]. Interestingly, in the green aphid, *Rickettsiella* usually coexists with the two symbionts, *Hamiltonella* and *Serratia*, which help the pea aphid evade parasitic wasps that are inclined to attack green aphids [24,100].

Our results showed that the gut microbiota of *B. germanica* was closely involved in defending against pathogenic fungal infection. Compared to the conventional German cockroaches, antibiotic-treated cockroaches were more susceptible to fungal infection [41]. The fecal extracts of conventional *B. germanica* had high inhibitory activity on the entomopathogenic fungus *Beauveria bassiana*, whereas, after antibiotic treatment, its fecal extracts lost the inhibitory activity. Some bacterial strains that can inhibit the growth of *B. bassiana* (*Bacillus atrophaeus*; *Bacillus subtilis*; *Pseudomonas reactans*) were successively isolated from the gut of *B. germanica* [33,40]. We isolated 13 bacterial strains with anti-*B. bassiana* activity; the strains isolated from foregut, midgut, hindgut, and feces of *B. germanica* occupied 23.1%, 30.8%, 15.4%, and 30.8% of 13 strains, respectively. An antifungal assay showed that the hindgut homogenate had the strongest anti-*Metarhizium anisopliae* activity. High-throughput sequencing further revealed that the composition of the hindgut microbiota was obviously different from those of other gut regions; higher diversity and abundance of *Bacteroides* and *Pseudomonas* were detected in the hindgut, which may be related to its strong antifungal activity [41]. These results account for the extremely low oral infection rate of pathogenic fungi.

In addition, the gut microbiota can interact with insect pathogenic microorganisms to accelerate host mortality. Broderick et al. found that the virulence of *Bacillus thuringiensis* to some insect larvae of Lepidoptera was closely related to their indigenous gut bacteria. Oral administration of antibiotics, which eliminated gut bacteria, reduced larval mortality caused by *B. thuringiensi*, whereas reintroduction of the bacterium (e.g., *Enterobacter* sp.) that normally resides in the midgut restored the insecticidal activity of *B. thuringiensis*, leading to the septicemia and insect death [101,102]. Moreover, Wei et al. discovered a crucial role of the gut microbiota in interacting with *B. bassiana* to accelerate mosquito death by downregulating the expression of dual oxidase and antimicrobial peptides in the midgut. Fungal infection can greatly increase the gut bacterial loads and reduce the bacterial diversity of mosquitoes, thus resulting in dysbiosis of the gut microbiota of mosquitoes. After fungal infection, the opportunistic pathogen *Serratia marcescens* overgrew and was transferred from the midgut to the hemocoel, thereby facilitating the fungal killing of mosquitoes [90]. It is speculated that the presence of gut microorganisms has a certain effect on the destruction of insects’ immune barrier, which suggests that disturbances caused by foreign toxin invasion may induce originally benign gut bacteria to exert pathogenic effects, thus allowing pathogens to infect insects more easily.

As insecticide resistance develops in the cockroach population [73,74,75], using pathogenic fungi as alternative tools to control cockroaches is urgently needed. Entomopathogenic fungi mainly infect insects through the cuticle. For indoor sanitary pests such as *B. germanica*, the toxic gel baits that are safe to humans seem to be a better alternative to surface applications for pest control. Entomopathogenic fungi *B. bassiana* and *M. anisopliae* are commonly used to control *B. germanica*, but they are seriously limited in practical application due to their low infection rate after passing through insect guts. Given the critical role of the gut microbiota of the German cockroach in defending against pathogenic fungi, we could try to disrupt or change the microecological composition of the cockroach gut with a synergistic agent such as bactericides (e.g., avermectins) or gastrotoxic preparation (e.g., boric acid), thereby increasing the oral infection rate of fungal insecticides. Moreover, the combined application of opportunistic pathogenic bacteria (e.g., *S. marcescens*) for oral feeding and pathogenic fungi (e.g., *M. anisopliae*) for topical infection could be a new strategy for synergistically controlling *B. germanica*.

## 6. Behavior: Interference with Aggregation and Trap Killing

Symbionts can synthesize or decompose some compounds to generate metabolites in insects, and certain metabolites are used by the host to synthesize pheromones or interspecific hormones, thus affecting the behavior of insects. The gut microorganisms such as *Lactobacillus plantarum* are able to influence the mating preference of *D. melanogaster* by changing the levels of sex pheromones. The fruit flies prefer to mate with individuals possessing similar gut microbiota [103,104]. The symbiont *Pantoea agglomerans* in the *Schistocerca gregaria* gut can utilize the digestion product vanillic acid from locusts to synthesize the aggregation pheromone precursor guaiacol, which promotes the aggregation of locusts [105]. Conversely, the microsporidian parasite *Paranosema locustae* can kill the gut microorganisms that are involved in the synthesis of aggregation pheromones through altering the immune system and intestinal chemical property of locusts, thereby preventing the swarm behavior of migratory locusts [106].

Cockroaches have a gregarious habit. Social interactions can help the individuals in many aspects of cockroach life such as providing greater protection from natural enemies, increasing access to resources, creating more mating opportunities, and even facilitating development and reproduction, which significantly improve survival fitness. Their aggregation behavior can also facilitate rapid and accurate foraging, even when facing frequently changing environments [107,108,109]. A recent study revealed that the gut microbes of *B. germanica* played a vital role in the biological synthesis of fecal volatile carboxylic acids (VCAs), a kind of pheromone that facilitates aggregation behavior. Forty kinds of highly attractive VCAs were detected in the fecal extracts of normal *B. germanica*, while only 28 kinds were detected in the feces of axenic cockroaches (gut microbiota removed). The axenic cockroaches inoculated with the aerobic bacteria from normal *B. germanica* feces recovered their aggregation behavior. The mixed inoculation of the six bacterial strains (*Enterococcus avium*, *Pseudomonas japonica*, *Weissella cibaria*, *Acinetobacter* sp., *Acinetobacter pittii*, and *Pseudomonas monteilii*) that were isolated from the feces of normal *B. germanica* strengthened the aggregation response of the axenic *B. germanica* compared to single inoculation [42]. This insight emphasizes the importance of symbiont-based strategies for cockroach control; we can artificially disrupt the aggregation behavior of cockroaches by interfering with the gut microbes related to behavioral modulation. The vitality of cockroaches would be significantly reduced if they lost their ability to gather, thereby further suppressing the cockroach population. Moreover, researchers have successfully prevented the gregarious behavior of locusts by utilizing microsporidian parasite *Nosema locustae* that can inhibit the growth of gut microbes associated with aggregation pheromone synthesis [106]. Similarly, if a microsporidian parasite can be identified to infect *B. germanica*, it could impact aggregation behaviors and reduce the fitness and survival rates of cockroaches, thereby achieving the aim of controlling cockroaches. In addition, we can further develop key bacterial strains or their active products into new attractants for pest control by using the characteristics of gut microbes with high attractant activity to host pests. A strain of *Hansenula anomala* was screened from the gut microorganisms of *Bactrocera minax*. Because its fermentation products had strong attraction to both male and female Chinese citrus flies, *H. anomala* was further developed as a microbe-derived attractant, which was 3.52 times more effective than traditional agents [110]. Therefore, we can isolate the microbes related to the synthesis of pheromones from cockroach guts and culture them in vitro to produce and extract aggregation pheromones which are combined with pesticides for cockroach control.

## 7. Paratransgenesis

Symbionts, especially gut microbes, are involved in most life activities of insects and play an important role in many physiological functions. In view of the close relationship between symbiotic microorganisms and insect physiology, a promising pest control strategy using transgenic symbionts has become a study hotspot. After gene modification in vitro, the insect symbionts can be used as a gene expression vector and be inoculated into the host; these engineered symbionts can express the effector molecules in hosts, thereby interrupting the transmission of vector-borne diseases. Compared with transgenic insects, transgenic symbionts are easier to be manipulated, expand culture, spread in the host population, and adapt to a wide range of species [111,112,113].

The parasitic protozoan *Trypanosoma cruzi* can cause Chagas disease, which is transmitted by the triatomid bug, *Rhodnius prolixus*. The gut symbiotic bacterium *Rhodococcus rhodnii* of the triatomine bug was genetically modified to secrete the anti-*Trypanosoma* protein and then was transferred into the gut of *R. prolixus* for inhibiting *T. cruzi* development, thereby greatly decreasing the carrying rate of *T. cruzi* [114]. The common symbiotic bacterium *Pantoea agglomerans* in the mosquito midgut was engineered to express anti-*Plasmodium* proteins such as the salivary gland and midgut peptide 1 (SM1), Scorpine, and four copies of *Plasmodium* enolase-plasminogen interaction peptide (EPIP) [(EPIP)4] utilizing the *Escherichia coli* hemolysin A secretion system to interfere with malaria development, thereby significantly decreasing the number of mosquitoes carrying parasites [112]. The insecticidal crystal protein gene *Cyt1A* was introduced into the gut symbiont *Enterobacter gergoviae* from the pink bollworm to be used for effectively controlling mosquito and blackfly larvae [115]. This suggested that genetically modified symbionts can be used as an effective tool for controlling pests and vector-borne infectious diseases.

Similarly, we could also use the paratransgenesis method to control *B. germanica*. The first step in the application of paratransgenesis is to find suitable symbiont candidates from *B. germanica*. After gene modification, transgenic symbionts would be reintroduced into cockroaches where they produce toxic effector proteins for cockroach control. Akbari et al. [116,117] found a potential candidate (i.e., *E. cloacae*) that could be used for paratransgenesis from *P. americana* midgut, which provides inspiration for the selection of symbiont candidates for the control of *B. germanica* via paratransgenesis. In addition, it has been confirmed that *E. cloacae* can be used as a vector of genetic modification for manipulating insect populations (e.g., *Phlebotomus papatasi* and *Glyphodes pyloalis*) [118]. Because the *B. germanica* midguts that were derived from the endoderm would not be damaged during ecdysis, the microorganisms colonized in midgut would be relatively stable throughout the life span of cockroaches, suggesting that the midgut of *B. germanica* might be a good site for selecting the appropriate transgenic symbionts [119]. However, more studies need to be done to find suitable paratransgenesis candidates from the midgut of the German cockroach; these symbiotic candidates can be cultured in vitro, can be vertically transmitted in host populations, have no pathogenic to humans, can be inserted with foreign toxic genes, and can be easily transferred into hosts. In addition, following dispersal of these transgenic symbionts into cockroach populations via feces for their coprophagy [109,120], the genes encoding insecticidal proteins would quickly spread throughout the population, thereby effectively suppressing the cockroach population in a self-sustaining manner. These engineered symbiotic bacteria might be a replacement for chemical insecticides, thus preventing the development of insecticide resistance and mitigating environmental pollution.

## 8. Conclusions and Prospects

With the development of molecular technologies such as high-throughput sequencing and metagenome analysis, the research bottleneck has been broken. Researchers can not only determine the classification and composition of symbionts in the host insect but also reveal their potential functions. The gut microbes of the cockroach are closely related to insecticide resistance and defense against entomopathogenic fungi. We can use an artificial gut system with cultured gut wall cells in vitro to simulate the cockroach gut to evaluate the interaction of the host insect, gut microbiota, and entomopathogenic fungi. In this way, we can obtain a better understanding of the symbiont–host interaction mechanisms and learn how to control cockroaches more effectively. To sum up, symbionts are common in cockroaches and are the guardians of the health and adaptation of host insects. They can provide nutrients for the growth and development of cockroaches, synthesize many bioactive substances, regulate the host’s immunity, and defend against predators and pathogenic microbial infection. Symbionts provide protection for host insects, such that the symbiotic combination has greater competitive advantages, which contributes to the stable maintenance of the symbiotic relationship. Finding the entry point of prevention and treatment from the cockroach–symbiont interaction must be considered as a promising method to effectively suppress the cockroach population. Some aspects of cockroach–symbiont interactions now have a firm experimental foundation, but others remain contentious. Future research should investigate the detailed mechanisms via which symbionts influence cockroach susceptibility to pathogens and insecticides; symbionts have the potential to be used as effective tools for cockroach control. In addition to mechanically carrying pathogens (e.g., *Pseudomonas aeruginosa*), cockroaches may also have a symbiotic relationship with some human pathogens and harbor some parasites (e.g., *Ancylostoma duodenale*, *Ascaris lumbricoides*), which would cause diseases in humans. Through studying the interaction mechanism between hosts and pathogenic symbionts, we can destroy their symbiotic relationships or utilize antagonism between symbionts to eliminate these human pathogens or parasites, thereby decreasing the vector capacity of cockroaches.

Through studying the interaction between the host and their symbionts, we are also able to learn more about the universal law of coevolution. The insect gut is a functionally diverse ecosystem that can efficiently transform poor, unbalanced, toxic, or indigestible substrates into substances for the insect’s own use. The knowledge of the mechanisms through which this happens could play a role in waste management, including the detoxification of contaminated substrates and the recycling of waste. However, in this context, the management of isolated strains would require more input, for both deepening research and developing applications.
